# Lacrimal sac rhinosporidiosis presented as nasal obstruction symptoms without any ocular manifestations: a case report

**DOI:** 10.1097/MS9.0000000000002898

**Published:** 2025-02-11

**Authors:** Manisha Paneru, Deepak Yadav, Dhirendra Yadav

**Affiliations:** a Department of Otorhinolaryngology, Patan Academy of Health Sciences, Laliptur, Nepal; b Patan Academy of Health Sciences, Lalitpur, Nepal

**Keywords:** lacrimal sac, mesomycetozoa, oculosporidiosis, rhinosporidiosis

## Abstract

**Introduction and importance:**

Rhinosporidiosis is a chronic granulomatous infection due to *Rhinosporidium seeberi* and it is endemic mainly in South Asian countries such as India and Sri Lanka. Though it commonly involves nasal and nasopharyngeal mucosa, the involvement of the lacrimal sac is rare. Diagnosis can be done by imaging techniques and confirmed on histopathology.

**Case presentation:**

A 22-year-old male presented with left nasal obstruction and an oropharyngeal mass, without any ocular symptoms. Imaging studies by CT scan revealed a mass involving the nasal and nasolacrimal regions. Biopsy confirmed rhinosporidiosis. The patient underwent a wide local excision of the mass with partial resection of the lacrimal sac. Postoperatively, the patient was given Dapsone for 6 months. Regular follow-up with nasal endoscopy and ophthalmology review showed no evidence of recurrence 1 year following surgery.

**Clinical discussion:**

Rhinosporidiosis typically presents as vascular polyps in the nasal or ocular areas. It is uncommon that there might be involvement of the lacrimal sac without any symptomatology relevant to the conjunctiva, which has been elaborated in the present case. Examination for the extent of disease is assisted by techniques such as CT and MRI. Histopathology is confirmatory, showing a typical appearance with sporangia filled with multiple endospores. Treatment can be carried out mainly by surgical excision supplemented by electro-coagulation to avoid recurrence. Dapsone post-operatively is recommended as recurrence is common in rhinosporidiosis.

**Conclusion:**

Although rhinosporidiosis predominantly affects the nasal mucosa, lacrimal sac involvement should be considered in the differential diagnosis. Surgical excision along with postoperative medication and regular follow-up forms the mainstay in the management of the disease to avoid recurrence.

**Highlights**

- Rhinosporidiosis can involve a lacrimal sac but is present without ocular symptoms as presented in our case.
- Diagnosis is done by confirming it through imaging studies and histopathologically, which shows sporangia with endospores.
- Surgical excision followed by postoperative Dapsone avoids recurrence.

## Introduction

Rhinosporidiosis is a chronic and granulomatous condition caused by *Rhinosporidium seeberi*, a class of mesomycetozoa, which is now considered to be a rare aquatic protistan parasite^[1,2]^. Previously, it was thought to be a fungus. This disease has been reported in over 70 countries, with India and Sri Lanka being the most affected^[3]^. The most common site of infection is the nose and nasopharynx, followed by the conjunctiva and lacrimal sac. In rare cases, other areas such as the lips, palate, trachea, vulva, penile region, rectum, and skin can also be affected^[4]^. The route of transmission is still debatable, but it is believed that the protozoa migrate through traumatized epithelium when they come in contact with contaminated soil or water. They complete their life cycle, resulting in polypoidal masses in the mucosal surfaces that tend to bleed. Clinically, rhinosporidiosis presents as a polyp, papilloma, or warty lesion that is soft, hyperplastic, and highly vascular^[5]^. The mainstay of treatment is surgical excision combined with electro-coagulation of the base of the lesion.

In this context, an unusual case of Rhinosporidiosis in the lacrimal sac is presented, which is presented without any ocular symptoms. This case has been reported following the SCARE criteria^[6]^.

## Case report

**Figure 1.**
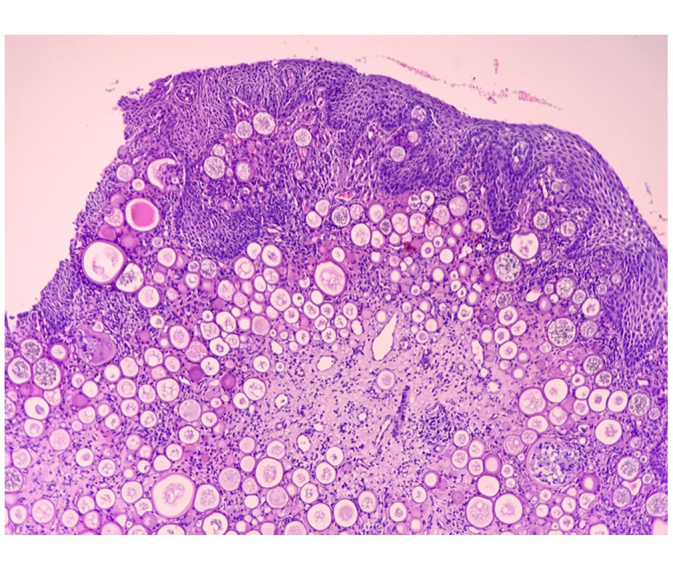
(a, b) Axial contrast enhance CT nose and nasopharynx showing heterogenous opacification in inferior turbinate and nasolacrimal duct respectively.

**Figure 2.**
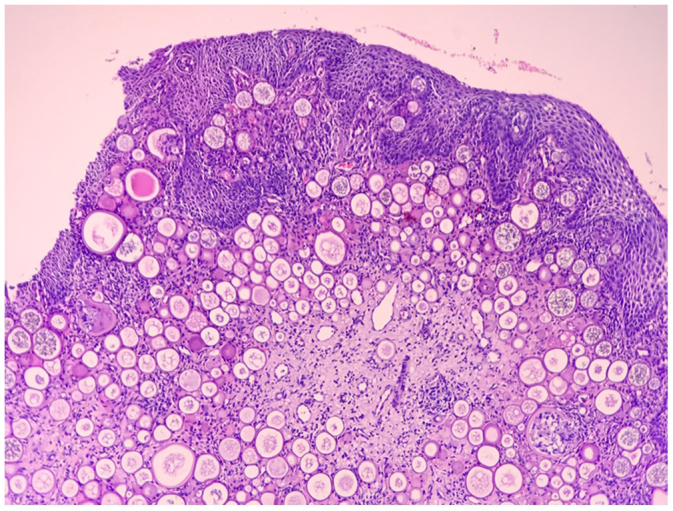
HPE slide showing mature sporangia with numerous spores.

**Figure 3.**
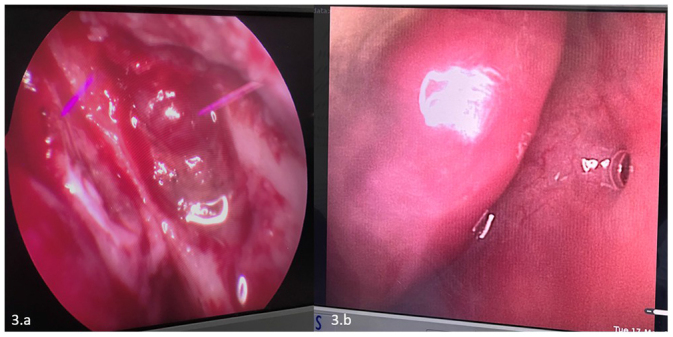
(a) 0-degree endoscopy showing Exposure of the lacrimal sac after removal of the frontal process of the maxilla. (b) Nasopharyngoscope normal mucosa, no signs of recurrence or scarring.

A 22-year-old male visited the OPD with complaints of left nasal obstruction and a mass hanging in the oropharynx for the past 5 months. The patient experienced nasal breathing difficulty and a sensation of a foreign body in his throat. The patient also experienced occasional bleeding from the mass hanging in the oropharynx, but no nasal bleeding, nasal discharge, epiphora, or diplopia. Upon examination, his general condition was good. On anterior rhinoscopy, there was decreased nasal patency and a deviated nasal septum towards the left side. Oral cavity and oropharyngeal examination showed a pinkish, polypoidal mass in the oropharynx (just behind the uvula). A contrast-enhanced CT scan of the nose and paranasal sinuses was performed, which revealed mild heterogeneous enhancement with the central vascular core and its attachment in the posterior end of the left inferior turbinate. The heterogeneous opacity was also noted in the posterior to nasolacrimal duct opening and is extending posteriorly into the choana and inferiorly into the oropharynx (Fig. 1a, b). A biopsy was performed under local anesthesia in ENT OPD, and histopathology showed mature sporangia with numerous spores, supporting the diagnosis of Rhinosporidiosis (Fig. 2). The patient underwent wide local excision of the mass with cauterization of the base under general anesthesia. Intraoperatively, the whole of the mass along with the posterior end of the inferior turbinate, the Hasner’s valve, the nasolacrimal duct, and the sac was opened and Partial resection of the lacrimal sac was done (Fig. 3a). The patency of the lacrimal apparatus was ensured by syringing intraoperatively. An ophthalmological review was done for the involvement of the lacrimal sac in the imaging, and syringing of the left lacrimal duct was performed, which showed blockage of the lacrimal passage. The postoperative stay was uneventful, and the patient was given Dapsone for 6 months. Monthly follow-up with nasal endoscopy and ophthalmology review was done (Fig. 3b), and there were no signs and symptoms of recurrences till 1 year of follow-up.

## Discussion

In 1900, Guillermo Sebber treated a 19-year-old farm worker in Australia who was suffering from unilateral nasal obstruction caused by a nasal mass, which he termed Rhinosporidiosis. Later in 1923, Ashworth described the life cycle of the organism and renamed it *R. seeberi* after observing its similarity to the fungal life cycle^[7]^. The prevalence of rhinosporidiosis is noted all over the world, in more than 90 countries, it is usually found in the tropics, being endemic in South India, Sri Lanka, parts of East Africa, and South America^[8]^. It has a male predominance and is usually seen between the second to fourth decades of life^[9]^.

Rhinosporidiosis typically presents as a sessile or pedunculated polyps and is occasionally surrounded by whitish spores giving a classical appearance of a strawberry-like growth. The clinical presentation is variable and usually depends on the site of involvement. The nasal symptoms could be nasal obstruction; occasional epistaxis along with nasal discharge and ocular involvement has symptoms like epiphora, redness in the conjunctiva, itching of the eyes, and photophobia. In cases of lacrimal sac involvement, it presents as an isolated and non-tender swelling over the medial canthus, for which dacryocystorhinostomy (DCR), via DCR stent.

The DCR stent plays a crucial role in maintaining the patency of the rhinostomy during the healing phase, preventing stenosis caused by fibrosis or granulation tissue formation. It acts as a scaffold, allowing for proper tear drainage while the surrounding tissue heals.

The term oculosporidiosis is given when this protozoan infection affects the ocular region and is seen in 10–55% of cases of rhinosporidiosis. The conjunctiva is the most commonly affected tissue (>70%) followed by the lacrimal sac (22%) with or without conjunctiva involvement^[10]^. The mode of transmission is still controversial, but the spread probably occurs through stagnant water and dust. Trauma is a necessary prerequisite for infection as it is the site for inoculation of the spores and its migration and completion of the life cycle^[11]^.

When the lacrimal sac is affected in isolation, it is believed that the infection reaches the sac from the nose or eye through the lacrimal canaliculi without affecting the nose or conjunctiva. Similarly, in the case report presented, there was involvement of the nose but not the conjunctiva.

Contrast-enhanced CT and MRI imaging can help diagnose and assess the extent of the disease^[12]^. However, a definitive diagnosis requires the examination of the biopsy specimen under the microscope. The oval-shaped sporangia, containing hundreds of endospores, are easily identified under the microscope and are a typical feature of rhinosporidiosis.

Surgical excision accompanied by electro-coagulation of the lesion’s base is the most effective treatment for minimizing the risk of recurrence. Postoperative dapsone treatment is also recommended to prevent recurrence, which can have severe complications, including life-threatening dissemination and secondary bacterial infection in the affected area.

Endoscopic DCR remains valuable even after partial or total resection of the lacrimal sac or duct, as it enables the creation of a direct anastomosis between the remaining lacrimal tissue or surrounding mucosa and the nasal cavity. This minimally invasive approach minimizes scarring and promotes effective drainage, particularly in cases where coexisting nasal pathologies are also present.

Pharmacological treatment, primarily with dapsone, serves as an adjunct to surgery in rhinosporidiosis, helping to reduce lesion size and prevent recurrence by targeting residual spores. While effective in lowering recurrence rates postoperatively, it is not curative on its own. Adverse effects include hemolysis (especially in G6PD deficiency), methemoglobinemia, hypersensitivity reactions, and gastrointestinal or neurological symptoms. Close monitoring is essential to balance efficacy and safety.

## Conclusion

To conclude, while rhinosporidiosis is a disease that primarily affects mucosal areas, it can also impact deeper structures, including the lacrimal sac. Therefore, it should be considered as part of the differential diagnosis for any cases with lacrimal sac pathology. Treatment mainly involves surgical excision, but trans-nasal endoscopic excision with a new drainage pathway between the lacrimal sac and the nasal cavity, bypassing an obstructed nasolacrimal duct (dacryocystorhinostomy) can also be attempted in cases with limited sac disease. However, further studies on endoscopic excision are necessary to establish its efficacy. Postoperative treatment with dapsone is essential to prevent recurrence, and regular follow-up is necessary as rhinosporidiosis recurrence is common.

## References

[1] Arseculeratne SN. Recent advances in rhinosporidiosis and *Rhinosporidium seeberi*. Indian J Med Microbiol 2002;20:119–31. 17657050

[2] Mishra L Gupta S Pradhan S. Lacrimal sac rhinosporidiosis. Plast Aesthetic Res 2015;2:353.

[3] Arias AF Romero SD Garcés CG. Case report: rhinosporidiosis literature review. Am J Trop Med Hyg 2021;104:708–11. 10.4269/ajtmh.20-0291 PMC7866367 33289469

[4] Almeida FA Teixeira-Junior AAL Pinho JD. Evaluation of diagnosed cases of eye rhinosporidiosis in a public hospital of Maranhão, Northeast Brazil. BMC Ophthalmol 2019;19:218. 31703563 10.1186/s12886-019-1223-x PMC6842159

[5] Ghosh A Saha S Srivastava A. Rhinosporidiosis – unusual presentations. Indian J Otolaryngol Head Neck Surg Off Publ Assoc Otolaryngol India 2008;60:159–62. 10.1007/s12070-008-0003-3 PMC3450519 23120527

[6] Sohrabi C Mathew G Maria N. The SCARE 2023 guideline: updating consensus surgical case report (SCARE) guidelines. Int J Surg Lond Engl 2023;109:1136–40. 10.1097/JS9.0000000000000373 PMC10389401 37013953

[7] Parida PK Thangavel S Raja K. Lacrimal sac rhinosporidiosis. BMJ Case Rep 2021;14:e243926. 10.1136/bcr-2021-243926 PMC8240581 34183318

[8] Ocular rhinosporidiosis – a case report. Indian J Clin Exp Ophthalmol. Accessed 19 September 2024. https://www.ijceo.org/article-details/16841

[9] Rhinosporidiosis: a chronic tropical disease in lateral pharyngeal wall – PubMed. Accessed 19 September 2024. https://pubmed.ncbi.nlm.nih.gov/26155503/10.7860/JCDR/2015/11831.5951 PMC4484095 26155503

[10] Mukherji P Shilpy N. Ocular rhinosporidiosis – a case report. Indian J Clin Exp Ophthalmol 2022;8:295–97.

[11] Blitzer A Lawson W. Fungal infections of the nose and paranasal sinuses. Part I. Otolaryngol Clin North Am 1993;26:1007–35. 8290280

[12] Imaging features of rhinosporidiosis on contrast CT – PubMed. Accessed 19 September 2024. https://pubmed.ncbi.nlm.nih.gov/24347850/10.4103/0971-3026.120267 PMC3843328 24347850

